# The process evaluation of *It's Your Move!*, an Australian adolescent community-based obesity prevention project

**DOI:** 10.1186/1471-2458-10-448

**Published:** 2010-07-30

**Authors:** Louise B Mathews, Marj M Moodie, Annie M Simmons, Boyd A Swinburn

**Affiliations:** 1 School of Education, Deakin University, Geelong, Victoria 3217, Australia; 2 Deakin Health Economics Unit, Deakin University, Burwood, Victoria 3125, Australia; 3 WHO Collaborating Centre for Obesity Prevention, Deakin University, Victoria 3217, Australia

## Abstract

**Background:**

Evidence on interventions for preventing unhealthy weight gain in adolescents is urgently needed. The aim of this paper is to describe the process evaluation for a three-year (2005-2008) project conducted in five secondary schools in the East Geelong/Bellarine region of Victoria, Australia. The project, 'It's Your Move!' aimed to reduce unhealthy weight gain by promoting healthy eating patterns, regular physical activity, healthy body weight, and body size perception amongst youth; and improve the capacity of families, schools, and community organisations to sustain the promotion of healthy eating and physical activity in the region.

**Methods:**

The project was supported by Deakin University (training and evaluation), a Reference Committee (strategic direction, budgetary approval and monitoring) and a Project Management Committee (project delivery). A workshop of students, teachers and other stakeholders formulated a 10-point action plan, which was then translated into strategies and initiatives specific to each school by the School Project Officers (staff members released from teaching duties one day per week) and trained Student Ambassadors. Baseline surveys informed intervention development. Process data were collected on all intervention activities and these were collated and enumerated, where possible, into a set of mutually exclusive tables to demonstrate the types of strategies and the dose, frequency and reach of intervention activities.

**Results:**

The action plan included three guiding objectives, four on nutrition, two on physical activity and one on body image. The process evaluation data showed that a mix of intervention strategies were implemented, including social marketing, one-off events, lunch time and curriculum programs, improvements in infrastructure, and healthy school food policies. The majority of the interventions were implemented in schools and focused on capacity building and healthy eating strategies as physical activity practices were seen by the teachers as already meeting students' needs.

**Conclusions:**

While substantial health-promoting activities were conducted (especially related to healthy eating), there remain further opportunities for secondary schools to use a whole-of-school approach through the school curriculum, environment, policies and ethos to improve healthy eating, physical activity and healthy body perceptions in youth. To achieve this, significant, sustained leadership will be required within the education sector generally and within schools specifically.

## Background

The prevalence of adolescent obesity continues to rise in many countries [1,2] and reversing this trend is a major public health challenge. In Australia, about one in four adolescents is overweight or obese [3], up from about one in ten in 1985 [4].

Adolescents are an important group to target for obesity prevention programs for many reasons: there is a high rate of tracking of obesity from adolescence to adulthood [5,6]; they are facing substantial further increases in overweight and obesity from age-related changes (currently over 60% in Australian adults) [7] and secular change; they are reasonably accessible through secondary schools, and; society has an obligation to provide healthy environments for children and adolescents. It is therefore surprisingly that there are so few adolescent obesity prevention studies in the literature [8,9].

It's Your Move! (IYM) was the Australian component of the Pacific OPIC (Obesity Prevention in Communities) Project - a three-year adolescent obesity prevention project in Australia, Fiji, Tonga, and New Zealand [10,11]. All projects aimed to reduce the prevalence of overweight and obesity using quasi-experimental designs [11,12]. Evaluating the process of any intervention program is critical for being able to understand the outcomes in terms: of whether the program was implemented as planned; what were the characteristics of the intervention in terms of dose, reach, adoption, fidelity and so on; what were the perceptions and responses of participants, and; what factors impeded or facilitated the program activities [13]. Process evaluations are particularly important for complex interventions because there will inevitably be substantial heterogeneity of program implementation and understanding this is essential to explaining the eventual outcome results.

This paper aims to evaluate the process of the IYM project by analysing how the project's objectives were met, identifying the barriers and lessons learned in the program delivery, and assessing how well the project met best practice principles for community-based obesity prevention [14]. These best practice principles were developed within the Australian context as a result of extensive reviewing of the literature and stakeholder consultation to define the ingredients of effective community-based intervention projects.

## Methods

### Project Structure

IYM used a capacity building approach to promote healthy eating, regular physical activity and healthy bodies amongst youth aged 13-17 years, and to improve the capacity of families, schools and community organisations to sustain the promotion of such messages [11]. The project was funded by the National Health and Medical Research Council and the Victorian Government (Department of Health) and was supported by Deakin University in terms of training, expertise and evaluation [10]. A Reference Committee, comprising school principals, stakeholders from the relevant government departments and Deakin University, provided higher level strategic direction, support and budgetary approval, and monitored progress. A Project Management Team, comprising a Project Coordinator (Louise Mathews) and the five School Project Officers (SPOs) supported by a Deakin University representative, managed project implementation. Within each school, the SPOs were appointed by the principal from the existing staff to coordinate implementation of the action plan objectives. Of the five SPOs, two were home economics teachers and the other three, health and physical education teachers. Each was allocated one day per week to work on the project for the three year period. The Project Coordinator also had a secondary school teaching background, coupled with postgraduate research studies.

### Guiding frameworks

A number of frameworks informed the project intervention and evaluation. The overall intervention was conceived as one of community capacity building since that gave a common intervention across schools yet with sufficient flexibility to meet the varying needs of the schools and participants. The New South Wales Framework for capacity building [15] was adopted later in the program and its domains (partnerships, leadership, resource allocation, workforce development and organisational development) were used as the structure to evaluate this aspect of the program. We have adopted this report's definition of capacity building as an approach to 'the development of sustainable skills, structures, resources and commitment to health improvement in health and other sectors to prolong and multiply health gains many times over'.

The formative evaluation used the ANGELO Framework (Analysis Grids for Elements Linked to Obesity) [16] since that was able to combine international and local evidence with a community participatory approach to develop an action plan in an efficient manner. A set of Best Practice Principles for community-based obesity prevention has recently been developed in Australia based on a review of the international literature and extensive community consultations [14]. While these were developed after the IYM Project, they represent the benchmarks against which Australian programs should be assessed and we used that framework to structure the discussion of the IYM process. They cover community engagement (methods, community analysis to inform planning, partnerships, community capacity); program design and planning (problem analysis, positioning and framing, planning context); implementation and feasibility (consumer testing; quality monitoring) and governance and accountability (explicit funding sources, project management structure).

### Participants

The selection criteria for choosing the intervention sites have been previously published [11]. The selection of the intervention communities was based on a number of criteria. The communities had to have sufficient numbers of youth to reach the sample sizes; sufficient numbers of settings (mainly schools, community organisations) to provide the structures for interventions; a degree of geographical cohesiveness to be able to define the sampling frame; and reasonable proximity to the intervention and evaluation teams.

Two samples, both the control and intervention, were drawn from the Barwon-South Western (BSW) region of Victoria. The BSW region is a broad geographical region of ~29,637 square kilometres in the south-western region of Victoria. It has an estimated residential population of approximately 350,109 people of which 249,301 are from one large catchment. There are 49 secondary schools (31 = government, 5 = catholic, 13 = private) with approximately 49,000 children enrolled in secondary schools. The intervention sample was drawn from all secondary schools from the East Geelong and Bellarine Peninsula regions of Geelong (n~4,500). Five schools with a total available sample n of ~3406) were selected. The comparison sample consisted of seven secondary schools and was similar in size and representative of schools from the Barwon-South Western Region. Demographically, 40.7% of students were in low SES areas and 56% of students were male. The mean age of the sample was 14.6 years. This demographic profile is reflective of the region. All students and their parents gave written informed consent covering their participation in the study.

### Design, development and implementation of the interventions

Before the project commenced, ethics clearance was obtained from Deakin University Human Research Ethics Committee and Victorian Department of Education. Initial project activities focused on the teachers and students from the five intervention schools and key stakeholders with five students (who were either approached or volunteered) and one teacher (selected by the Principal) from each school participating in a two day ANGELO (Analysis Grid for Elements Linked to Obesity) workshop. This was facilitated by the university's support and evaluation team. Participants were guided through a process to set priorities for action, culminating in the formulation of a 10-point action plan [10,11].

At baseline (response rate = 51%) and follow up (response rate = 68%), students completed a knowledge, attitude and behavioural survey about their nutrition and physical activity patterns and had anthropometry assessments [17]. The outcome measures were relative changes in anthropometry (body mass index z-score), body fatness (measured by bioelectrical impedance) and quality of life [11]. In addition, before and after assessments of the school and community environments were made using a three-part school environmental audit administered to the principal, canteen manager and three teachers and a Community Readiness to Change Index [18]. The proposed intervention, mediators, moderators and outcomes were built into a logic model which guided the conceptualisation of the intervention effects [19]. Preliminary analyses of these data were used to further inform the development of interventions although in reality, these analyses only became available part way through the intervention period and thus served to refine rather that fundamentally change the action plan implementation.

Within the schools, the project targeted the whole-of-school, working with principals, teachers, canteen managers and business managers, as well as students and parents. The Project Coordinator worked collaboratively with the SPOs to design and develop school-specific intervention activities, under each of the ten broad objectives of the action plan. The SPOs, who were teachers released from teaching duties one day per week (using project funding to buy their time), were encouraged to take ownership of the project and to implement sustainable interventions within their school's environment, structure and curriculum. Each SPO selected or invited students to apply to become Student Ambassadors whose role was to champion the project within their particular school.

### Process evaluation methods

A data collection proforma covering data about intervention activity process (i.e. how the activity was conducted), dose (i.e. scale/duration of the activity), frequency (i.e. how often the activity/event was conducted), reach (i.e. how many/type of people involved - including both adolescents and members of the wider school community) and associated resource use (i.e. for the subsequent economic evaluation) was developed, and completed by project personnel for all activities related to intervention planning and delivery. The data were entered into an ACESS database and some 1,400 entries were recorded throughout the three year duration of intervention activities. Entries were aligned with one objective which meant in collating the results; actions were only counted once so that the analysed data which are reported on in the tables are mutually exclusive. These process evaluation data were supplemented by information drawn from the IYM implementation reports on each of the 10 strategic objectives (available in reports at http://www.deakin.edu.au/hmnbs/who~obesity), which include formal and informal notes recorded by the project staff at meetings, and correspondence and communication (emails and telephone calls) with personnel involved in intervention delivery. Interviews were conducted with 65 Student Ambassadors in 2007-2008, and the five SPOs in 2005 and 2008.

## Results

### The action plan

The 10-point action plan (Table 1) consisted of three guiding objectives (Capacity Building: Objective #1, Social Marketing: Objective #2, Evaluation: Objective #3), four nutrition objectives (water versus sweet drinks: Objective #4, breakfast: Objective #5, fruit and vegetables: Objective #6, and food at school: Objective #7) and two physical activity behavioural objectives (active transport: Objective #8, and organised sport: Objective #9), and an innovative objective which focused on the promotion of a health body image as a priority for adolescents (Objective #10). The formative evaluation (Objective #3) component to develop the action plan has been described elsewhere [10,16].

**Table 1:** It's Your Move Action Plan outline

Objectives	Key Strategies
1. To build the capacity of families, schools, and community organisations to promote healthy eating and physical activity	Identify resources Develop and maintain the necessary structures and relationships Provide ongoing training for students, staff & others Develop the programs, policies & activities

2. To achieve a high awareness of the projects key social marketing messages	Develop and implement a social marketing plan

3. To evaluate the process, impact and outcomes of the It's Your Move! Project	Formative evaluation Process evaluation Impact and outcome evaluation Dissemination

4. To significantly decrease the consumption of high sugar drinks and to promote the consumption of water	School canteen/vending machine policies Curriculum Parent information

5. To significantly increase the proportion of young people eating breakfast	Promote time management skills for young people Parent information and motivation

6. To significantly increase fruit and vegetable consumption	Canteen availability/promoting/pricing of fruit and vegetables Programs and activities Parent information on fruit and vegetables

7. To significantly increase the healthiness of school food	School food policies Canteens (availability, promotion, pricing)

8. To significantly increase active transport	Parent information School Policies (drop-off zones etc)

9. To significantly increase participation in organised sports and other active recreation	Parent education (support, role models) School policies on participation Change school rules/systems to support facility/equipment use Partnership programs with clubs

10. To create an acceptance of different healthy body sizes/shapes and decrease episodes of inappropriate dieting	Develop social marketing messages with students to promote healthy bodies in all shapes and sizes, and discourage inappropriate 'dieting' practices Curriculum integration

In the following sections, each strategic objective (or group of objectives) is discussed. A table is provided for each (Tables 2, 3, 4, 5) which documents associated activities and their dose, frequency and reach.

**Table 2:** Activity summary for capacity building (Objective #1)

Category	Description	Objective number (#)	Distribution and comments
**Resources**			
Funds	$12,000 secured from five competitive grants	#1	Funds from local businesses for Student Ambassador training
Personnel	1 Project Coordinator	M	(0.8 EFT year 1, 0.5EFT year 2, 0.2 EFT year 3)
	5 SPOs	M	(0.2 EFT years 1-3)
Materials	125 pedometers	#8 #9	Support walking programs

**Leadership**			
Students	65 Student Ambassadors	M	2 groups selected across five schools (2005/06 [25 students] and 2007/08 [40 students])
	60 presentations conducted by the Student Ambassadors	M	Inform teachers and students of events and activities
Other	120 presentations SPOs their school community	M	Provide information about project events and activities

**Workforce development**			
Students	Completed tertiary certificates (8 day training course)	#1	40 Student Ambassadors
	1/2 day media training course	#2	20 students (non Student Ambassadors)
Other	Attended 1 day professional development training	#2	4/5 canteen managers
	Project Coordinator attended (2 × 5 day short courses)	#1, #2	Health promotion and obesity prevention short courses
	Project Management Team attended 2 day social marketing course	# 2	Project Coordinator and 5 SPOs
	3 × 90 minute Physical Education Teachers Professional Development sessions conducted	#1, #9, #10	105 teachers attended more than one session (badminton, touch rugby, minor games)

**Partnerships and collaborations**			
Government	Member of the Regional Network of Primary Care Partnership	#1	Project coordinator attended 6 × 2hour meetings
	Member of the local youth needs study	#1	Project coordinator attended 6 × 2 hour meetings
NGOs	Collaboration with Leisure Networks	#8, #9	On physical activity objectives
Others	Collaboration with regional newspaper to develop recipe books	#5, #6, #7	200 students involved; 150,000 recipe books distributed with local paper over two week period

**Organisational development**			
Schools	Changed catering practices for school and staff functions	#4, #5, #6, #7	5/5 schools improved food service requirements for catering
	Colour coding canteen menu	#4, #5, #6, #7	5/5 schools implemented traffic light colour codes to their canteen menu

#, number; M, multiple objectives and/or strategies; EFT, effective full-time; SPO, School Project Officers; NGOs, non-government organisations

**Table 3:** Activity summary for social marketing objective (Objective #2)

Category	Description	Objective number (#)	Distribution and comments
**Media reports and promotions**			
Television	One 5 minute item (repeated twice)	M	Within a 30 minute program on healthy lifestyles on national TV channel
Print	8 newspaper articles	#1(4), #4(2), #6(1), #7(1)	Regional newspaper (distribution = 30,000 copies daily)
Radio	1 × 10 minute interview with 7 Students Ambassadors and 1 SPO	# 11	Local radio (Geelong)

**Developed materials**			
Printed	8 posters	#1-7 (1 each)	A3 posters for schools (total n = 105); developed by students
	8 sets of stickers	M	For student diaries (total n = 250)
	6 project newsletters	M	Developed by Project Coordinator and SPOs distributed to project network (n = 60)
	Water bottle postcard	#4	125 A5 sized postcards
	Water bottle rules	#4	125 A4 sized rules
	Safe Food Handling @ School Guidelines*	#7	125 A4 sized food handling rules
	150 items placed in school newsletters - by SPOs	M	Fortnightly newsletters in 2/5 schools distributed to parents
	130 Certificates of Appreciation awarded to Student Ambassadors	M	Recognition of contribution
Other	1 project banner	M	For display at 10 events (conferences and in-school events)

**Sourced materials**			
Printed	2600 water bottles distributed during the project	#4	Provided to teachers and students early in the project through events, school booklists and the canteen
Other	5/5 schools involved teachers to develop curriculum units to design posters for each objectives	M	Art and Graphics teachers recruited to support the development of social marketing materials as classroom activities

#, number; M, multiple objectives and/or strategies; EFT, effective full-time; SPO, School Project Officers:

* Safe food handling - pamphlets designed to inform students of safe food handling practices in the classroom in line with the Department of Education/Food Handling Act

**Table 4:** Activity summary for nutrition objectives (Objective #4, #5, #6, & #7)

Category	Description	Objective number (#)	Distribution and comments
**Policies**	1/5 schools implemented a water policy	#4	
	5/5 developed a whole school food policy	#4, #5, #6, #7	

**Programs**	3/5 schools developed curriculum	#4, #5, #6	SPOs developed a new unit to teach in Health Education classes (8 lessons unit year 7-9 health education)
	1/5 schools conducted a breakfast program 1 × week over two years (n = 20 students each week)	#6	SPO and Wellbeing coordinator provided cereal and fruit to students before school
	2/5 conducted apple slinky programs	#7	Two schools rotated machine for one week per class over two years

**Activities**	2/5 schools developed advertisements in drama/media classes	#4	Assignment conducted over two terms (26 weeks). 4 × 30 second advertisements developed (n = 15 students involved)
	5/5 schools conducted 'one off' healthy eating days	#6	65 one-off health eating days
	Parent evening with a leading nutritionist (13 parents, 5 SPOs, 1 project coordinator)	#4, #5, #6	1.5 hour presentation and discussion about adolescent healthy eating practices in the home

**Infrastructure and Equipment**	4/5 schools installed new water fountains	#4	10 new fountains
	5/5 schools reduced, completely removed or changed the contents of vending machines	#4	Removal of all sweet drinks
	3/5 constructed a vegetable garden	#7	Produce used in cooking classes
	5/5 purchased food preparation equipment for their school	#7	Purchased equipment for - canteen and home economics classrooms

#, number; M, multiple objectives and/or strategies; SPO School Project Officer

**Table 5:** Activity summary for physical activity objectives (Objective #8, #9)

Category	Description	Objective number (#)	Distribution and comments
**Policies**	0/5 schools implemented new physical activity policies	#8, #9	5/5 schools had written guidelines and expectations outlined in the school diary prior to the project

**Programs**	1/5 *Walk 2 Where? *Curriculum*	#8, #9	Classroom development activities and assignment (2 hours × 26 weeks × 25 students)
	1/5 Walk 2 Where? Whole School Walking Activity	#8, #9	Whole school walking program (1 term × 1,000 students × 1 year)
	5/5 schools piloted the integration of pedometers into the classroom package	#8, #9	Classroom activities
	5/5 conducted lunchtime programs	#8, #9	231 hours of programs conducted over 3 years - walking groups, dance, martial arts, basketball, soccer and yoga

**Activities**	1/5 schools conducted the annual 'Ride 2 School' day (1 per year)	# 8, #9	Link with Bicycle Victoria 'Ride 2 School Program' (n = 70 students & 10 staff × 2 days per year in 2007 & 2008)
	4/5 schools had the 'Go for your life' mobile education unit** attend their school (1 day × 1 year)	#8, #9	Conducted with junior secondary students (n = 720)

**Infrastructure and Equipment**	1/5 schools received funding to built a new bicycle storage facility	#9	Provide shelter and security for staff and students bicycles

#, number; M, multiple objectives and/or strategies

* Walk 2 Where? - a whole school walking program designed by students.

** Go for your life mobile education unit -an external program run by the Department of Human Services to educate students on healthy eating and physical activity practices

### Capacity building

The aim of the Capacity Building (Objective #1) was to build the capacity of families, schools, and community organisations to promote healthy eating and physical activity, although in reality, these efforts centred predominantly around schools. This objective drew upon resources (financial and human resources, specialist advice) to: assist with the implementation of other objectives in the action plan; promote leadership in the form of teacher and student 'champions' within the schools to drive the implementation process (mainly through the Student Ambassador Model); provide workforce development for both students and teachers; create partnerships to link with both existing and emerging projects and organisations within the region; and work with the schools to incorporate healthy eating and physical activity policies and practices into their school charter. Table 2 provides a summary of the activities for capacity building (objective #1).

#### Resources

The project management team were successful in obtaining small amounts of funding through local community grants. This funded formal training for SPOs and Student Ambassadors.

#### Leadership

The implementation processes were greatly enhanced by the fact that all project staff had an educational background, and knew how to work with adolescents and to develop a pathway whereby young people could be provided with training to design and deliver interventions. The Student Ambassadors Model was constructed around 5-8 students per school who were given formal training in health promotion/event management by the local Technical and Further Education College, and then applied these skills to the project task to which they were assigned. Students were selected by teachers on the basis of their perceived motivation, and the number of ambassadors per school was necessarily kept small, which resulted in no drop-outs or ambassadors who were later viewed as poor selections.

#### Workforce development

The opportunities to provide additional professional development sessions for the SPOs and teachers were limited, given the already heavy additional roles and responsibilities which teachers had on top of their normal teaching commitments (e.g. sports supervision and out-of-hours meetings etc).

#### Partnerships and collaborations

The project linked in with a number of government and non-government agencies operating in the region. For example, the development of a Physical Education Professional Development Network (as part of objective #9) was piloted by a local sporting agency, and provided teachers with the opportunity to attend quarterly sessions where they could learn or refresh their skills in particular sports/activities. However, in the longer term, the agency was unable to sustain the model given their limited staff resources and the face-to-face support which teachers required to maintain the network.

#### Organisational development

There were varying degrees of support afforded to the project across the five schools. Where policy or system changes were undertaken in the school, the principal was closely involved in supporting and directing the changes at an organisational level. Having this higher level of support was fundamental to obtaining project 'buy-in' from both teachers and students.

#### Lessons learned

• The Student Ambassador model promoted student ownership of the project.

• School principal support is fundamental to achieving buy-in by both teachers and students.

### Social Marketing

Objective #2, social marketing (Table 3) had two phases. Phase I related to the first half of the project (2005-2006) and focused on awareness-raising and promoting the project to other staff in each school. This phase was time-intensive for the Project Coordinator and the SPOs who continually promoted the project in different forums, such as staff meetings and school and form assemblies. Phase II (2006-2007) focused on the production of targeted messages and resources for each of the behavioural objectives through work in the art curriculum. All five schools were able to utilise their art teachers and subjects to assist in the development of social marketing materials as part of normal classroom activities. The social marketing messages that appealed to students were those driven by the students, in terms of both conceiving the messages and preparing the associated marketing materials such as posters, brochures etc.

As demonstrated in Table 3, a major challenge for this objective was obtaining media exposure beyond the intervention sites, that is, beyond the school setting. In part, this can be attributed to the intervention being conducted in a regional area of Victoria and a lesser priority attached to the promotion of the project at that level by the Project Management Team. Most of the social marketing initiatives were focused at the school level, yet where action was taken at a wider level (such as the recipe books in the regional press), the project reach was broadened to include the harder-to-reach groups in the school communities such as parents.

#### Lessons learned

• Social marketing is fundamental to the project success, but is time and resource intensive.

• Extension of project reach to parents and the wider community necessitates different forms of social marketing strategies.

### Evaluation

The extensive evaluation (Objective #3) components conducted as part of this project were acknowledged by the schools as fundamental to the assessment of the effectiveness of such an approach. At a more practical level, the data collection requirements were continuous throughout the project, and included process evaluation, impact and outcome evaluation, economic evaluation and community readiness evaluation data reported elsewhere [7,12]. Members of the Project Management Team were responsible for facilitating data collection activities in the schools (such as the organisation around the taking of anthropometric measurements), in addition to supporting the process evaluation through the documentation of intervention events through an intervention activity log.

#### Lessons learned

• Evaluation is expensive in terms of time and resources, and requires an adequate allocation of dedicated funds.

• Evaluation and intervention planning and delivery roles should be assigned to different personnel to avoid tasks competing for time.

### The nutrition objectives

The four nutrition related objectives (Objectives #4, #5, #6, #7), (Table 4) focused on the promotion of water (versus sweet drinks), breakfast, fruit and vegetables, and food at school. Across these four objectives there was a mix of 'upstream' and 'downstream' strategies that ranged from 65 one-off healthy eating days, weekly breakfast programs, and the installation of new water fountains through to the development and ratification of a healthy eating (Food@School) policy which included guidelines for healthy eating in the curriculum, environment, canteen and school events.

The healthy eating days (such as soup days, salad days, sushi days) featured strongly amongst intervention activities. Staff and students reported that they enjoyed these designated healthy eating days, and they were perceived to be successful events by the SPOs, based on the amount of sales on the day. However, these activities were ultimately considered to be unsustainable given the additional resources required to implement them.

*Policy: *All schools designed a policy based on the 13 action areas defined by the 'Food@School' resource developed by the Project Coordinator, SPOs and the Reference Committee. The action areas included broad guidelines on the school food environment, canteen, curriculum, extra-curricula activities and events. There were a number of challenges with the implementation of the 'Food@School' action areas including the financial costs associated with effecting changes in the canteen, the lengthy consultation process required to gain support from the staff, parents, students and school council for the ratification of the policy, and limited time that the SPOs had to coordinate such a process.

High-level support is fundamental to the introduction of policy changes within schools and a good example of that was encountered in the project with the successes achieved by the water versus sweet drinks objective being enormously boosted by the Victorian Government's directive (during the course of the project) for the removal of sweet drinks from government schools.

#### Lessons learned

• One-off activities are neither sustainable nor have any lasting impact.

• Activities build into the school curriculum are effective, but require advanced planning and integration.

• Policy initiatives are effective, but require wide consultation and both 'top-down' and 'bottom-up' approaches.

### The physical activity objectives

Across the five schools there were a variety of programs and activities organised by the SPOs as part of the physical activity objectives to promote walking and cycling and getting active ([Objectives #8, #9], Table 5). One-off activities featured highly with 231 hours of organised lunchtime physical activities recorded across the three year period (over and above normal lunchtime activities).

There was reluctance from the schools to introduce new physical activity policy given that defined processes were already in place to deal with non-participators in physical education and sporting events.

Initiatives to promote active transport to and from school gained little traction in the schools given the regional catchments of some of the schools, their 'out-of-town' location, and the heavy reliance on bus transport. The implementation of active transport activities such as changing the drop off zone did not occur due to a high proportion of students who travelled to school by bus, concern about the weight of school bags and additional time it would take for students to walk the extra distance if dropped off a short distance from the school.

#### Lessons learned

• Active transport initiatives need to be cognisant of the geographical context of the school and its students.

• The introduction of policy initiatives around physical activity is difficult given a perception within schools that adequate structures already exist.

### The body image objective

The final objective (Objective #10) of the action plan, known as 'healthy body weight, shape and size', did not feature significantly throughout the project. This was primarily due to the sensitivities that existed around this topic and the depth of expertise required to effectively address the objective. There was only one major event conducted specifically for this objective - it was in conjunction with the Victorian Government's 'Go for Your Life' *Fad diets won't work *campaign which aimed to address the issue of inappropriate dieting practices among adolescents. As part of the event, approximately 500 students and 20 staff from the five intervention schools attended a one hour presentation, lead by a panel of four experts. The core of the presentation focused on debunking a number of dieting practices, and providing tips for healthy eating habits and messages for developing a healthy body weight, shape and size. Further to this, the SPOs of each school collaborated with their Health and Physical Education Coordinators to assist with the integration of this objective into the health curriculum. Curriculum modifications were made by all the intervention schools, the aim being to move body image away from the traditional focus on media-generated stereotypes to a more holistic understanding and acceptance of one's own body. Teachers reported that body image initiatives should target particular age groups, gender or specific peer groups than through mass one-off events.

#### Lessons learned

• The tackling of sensitive issues such as body image requires specialist staff.

## Discussion

The IYM project implemented a large number of activities and initiatives over the three year period. These are discussed using the structure from a set of 'Best Practice Principles for Community Based Obesity Prevention Initiatives' developed for the Australian context: community engagement; program design and planning; evaluation; implementation and sustainability [14].

### Community Engagement

This project linked five schools of varying educational contexts (government, religious and independent) to work collaboratively on this adolescent obesity prevention initiative. Once the principals formally committed their schools to the project, teachers and students were invited to be part of the engagement processes associated with the design and implementation of healthy eating and physical activities in their school. The student and teacher involvement in the formative stages of this project (consultations and action plan development) was a critical factor in gaining access to the school and leading to further capacity building approaches such as the development of the Student Ambassador program. The student engagement in the project was felt to be integral to the overall success of the activities and programs and also to the delivery of the social marketing messages. The Student Ambassador Model has the potential to be a positive, ongoing, high impact model due to the enjoyment derived from performing the role substantiated by benefits to others and themselves which included organisational, leadership and personal skills (public speaking, confidence) acquired and unexpected opportunities which arose such as employment. Further skills which the students acquired were in budgeting, prioritising ideas, attending meetings, developing timeframes and working with staff and students. However, to be sustainable, it needs to be structured so that the positive outcomes for both the students and the schools (development of leadership and health promotion skills) are maintained, while the potential negative influences (the time involved and the distraction from other school work) are minimized, and the costs are kept at a sustainable level (perhaps through local sponsorship arrangements or structured to be incorporated into other student roles such as school council membership or existing school leadership programs).

A major challenge for this project was to extend the project reach beyond the student community and to increase the knowledge and skills around healthy eating and physical activity to parents. Information was regularly placed in school newsletters, but face-to-face contact with parents was limited given the typically low level of involvement of Australian parents in the school life of secondary school students (relative to that of preschool and primary school parents), competing demands on their time and the regional nature of the school catchments. It was not possible to attract parents to project-specific evening information sessions. For example, an evening with Australia's most well-known nutritionist was conducted and only 13 parents attending the event. Project staff noted the amount of time and effort it took to organise such an event and felt that activities needed to be incorporated into those school occasions already built into the school calendar (such as parent-teacher nights, sports carnivals etc), as well as providing support for existing projects being conducted in schools and linking in with organisations whom have established connections with the school. The importance of interaction with families and the home environment to the success of obesity prevention programs is acknowledged, and it was made more relevant in the IYM project given the low socio-economic status of many of the participating communities. However, socio-economic status and the often greater preoccupation of parents with more basic issues of everyday life (maintaining a job etc) only exacerbated the problems of trying to secure their engagement.

#### Lessons learned

• Community engagement is important in developing collaborative partnerships amongst stakeholders so that the interventions are supported.

• Engagement of the wider community, and in particular, parents, may prove problematic in adolescent-focussed studies.

### Program design and planning

Secondary schools are complex organisations with many competing programs operating through the curriculum, lunch time activities and other initiatives targeting students and staff at any one time. This makes the incorporation of any new activities a challenge, especially if they are only peripherally related to educational outcomes. For the IYM project, the intensive evaluation requirements also substantially added to that challenge - indeed, the first point of contact for the students with the project was the taking of anthropometric measurements and filling in the baseline questionnaire.

Additionally, at a local school level there was no formal capacity building framework utilised in the project. In retrospect, the capacity building component of the project would have been enhanced with the adoption of a more formal and structured approach from the outset of the program. In addition to generic capacity-building activities, capacity building strategies were also integrated into all of the behavioural objectives of the action plan.

In planning to roll out the action plan, the Project Management Team held a number of planning days to brainstorm potential strategies under the different objectives. This provided for a sense of collaborative development, whilst at the same time, allowed each SPO to develop a personalised approach to suit the structures, teachers and curriculum of their specific school. For example, for the water objective, three schools focused on increasing the number of water fountains, whereas the other two did not identify that as a need.

#### Lessons learned

• The capacity building component of an intervention project needs to be formalised and structured at the outset.

• Effective program design and planning requires timely availability of baseline data to inform the interventions.

### Evaluation

This project suffered at the commencement of the intervention period from the high level of organisation required to coordinate the evaluation component; this often diverted the Project Coordinator and SPOs away from the implementation of interventions. Ideally, intervention projects should build in sufficient lead time for the collection, analysis and dissemination of the baseline data so that it can be used to inform the interventions. This project pursued a policy whereby the results of all data collection in the schools were fed back to the schools before being published and presented to the academic community.

### Implementation and sustainability

In the initial action plan presented to the Project Management Team, there were 27 strategies associated with the 10 objectives. The number of strategic actions associated with each of the 10 objectives varied considerably as did the take-up of actions by individual schools given their relevance and the school's level of interest and commitment. For example, the largest number of actions was under the heading of 'Water versus sweet drinks' given that this was high on the state government agenda at the time, and schools were keen to promote awareness and change the school environment around beverage consumption. Current practice in schools varied, and also a school might choose a different pathway (involving fewer strategic actions) to achieve the same strategic objective. For example, the introduction of a new policy into schools around the use of water bottles in the classroom varied from being a relatively quick and simple task in some schools where there was senior level support, whilst in others, it necessitated a wide range of activities to gain stakeholder commitment.

Upon completion of the project, the Project Management Team reported that only 16 (or 3.9%) of the 413 strategic actions were either not fully achieved (4) or started either (12) due to a shortage of intervention time, the complexity of a particular strategy and an inability to integrate it into school structures or curriculum (Table 6). All of the designated strategic actions were achieved for 7 of the 10 objectives, the exceptions being evaluation (11), food at school (4) and physical activity (1) (Table 6).

**Table 6:** Strategic actions achieved

	Objective 1 Capacity Building	Objective 2 Social Marketing	Objective 3 Evaluation	Objective 4 Water vs Soft drinks	Objective 5 Breakfast	Objective 6 Fruit and vegetables	Objective 7 Food at School	Objective 8 Active transport	Objective 9 Physical activity	Objective 10 Body image	All objectives	%
**Achieved**												
Across all schools	65	11	25	8		12	18		12	19	170	41.2
School 1				15	2	5	4	2	4	5	37	9.0
School 2				20	5	5	7	2	5	5	49	11.9
School 3				17	2	5	7	2	5	5	43	10.4
School 4				25	5	4	7	2	4	5	52	12.6
School 5				20	2	5	7	2	5	5	46	11.1
***Total***	**65**	**11**	**25**	**105**	**16**	**36**	**50**	**10**	**35**	**44**	**397**	**96.1**

**Partly achieved/not started**												
Across all schools			11								11	2.7
School 1											0	0.0
School 2							1				1	0.2
School 3							1				1	0.2
School 4							1		1		2	0.5
School 5							1				1	0.2
***Total***	**0**	**0**	**11**	**0**	**0**	**0**	**4**	**0**	**1**	**0**	**16**	**3.9**

												
***Total strategic actions***	**65**	**11**	**36**	**105**	**16**	**36**	**54**	**10**	**36**	**44**	**413**	**100.0**
***% all strategic actions***	**15.7**	**2.7**	**8.7**	**25.4**	**3.9**	**8.7**	**13.1**	**2.4**	**8.7**	**10.7**	**100.0**	

An examination of the activities and programs implemented by the project indicates that the majority of those conducted early in the project were in non-sensitive and non-contentious areas which created greater support for the project, than would have occurred if policy change had been immediately targeted. For example, as part of objective 4 to reduce sweet drink consumption, new water fountains were installed and IYM water bottles were provided to the staff and the Ambassadors, encouraging them to act as role models. In the second year, the Project Management Team decided that it would be more time-efficient to address the action plan by incorporating the healthy eating and physical activity strategies more closely into the curriculum. A more curriculum-focused approach allowed for the repetition of the messages and repeated 'doses' of intervention, as well as the building of a stronger connection between the awareness raising activities, canteen changes, and the healthy eating policies. Most of the SPOs chose activities and program areas which reflected their skills and experience (e.g. home economics teachers were more likely to focus on healthy eating rather than physical activity initiatives).

#### Curriculum

There were a number of challenges to the successful introduction of new curriculum developments. These included: finding space in the already-overcrowded curriculum in Australian schools; the lead time needed to plan and develop the curriculum (at least 6 months); the need to integrate the intended activities into the required assessment for that year level, and; teacher willingness to take on new areas within their subjects. To overcome these issues, the Project Coordinator and SPOs regularly met with the teachers and tried to make curriculum change easier for them by providing resources and advice around the new content or activity. Where appropriate, teachers were openly receptive to taking on objectives of the action plan such as the demonstration of sugar in sweet drinks within a health subject, and the development of a walking program and designing the associated social marketing messages within art classes. Having regular and adequate classroom time was crucial for students to research and complete the required assessment tasks and to obtain a greater understanding of the information underpinning the messages.

In relation to physical activity, there were no before-school or after-school activities arranged because a high proportion of students took school bus transport to and from school. Additionally, lunchtime activities were sporadically attended by students as they considered lunchtime as a time to catch up with friends.

#### Policy

Whilst the SPOs saw a need for policy around physical activity there was no support for such a policy from school management as there were already effective processes in place to deal with non participators in physical education and sport activities. In terms of healthy food provision, some principals and teachers believed that to achieve the desired outcomes associated with the implementation of a healthy eating strategy, policy needed to be driven beyond the school level by the State Government. At the outset of the project there was no state-level support for healthy food policies, but these were incrementally introduced during the course of the project, greatly facilitating the development of school-level policies. Within the school context, it is the principal and senior staff who need to provide the leadership to drive and monitor changes in policy because teachers are more comfortable with teaching approaches than developing and progressing policy through the school governance structures.

#### Lessons learned

• The implementation of intervention strategies need to consider their lasting impact on the environment and behaviours.

• A program needs to be multi-faceted and include both policy, curriculum and activity initiatives to ensure student and school engagement, commitment and sustainability.

### Governance and accountability

Structurally, the project commenced with an interim steering committee comprising school principals, key stakeholders and members of the university support and evaluation team. This committee focused on establishing the formative components of the project, roles and responsibilities of the project personnel and obtaining agreement to the terms of reference for the forthcoming intervention period. Once the intervention period commenced, this committee was revised into two components: a Reference Committee (for advice, monitoring and strategic directions) and the Project Management team (for project implementation). The communication between the two groups, managed by the Project Coordinator, was transparent, with the minutes of meetings and various discussions documented and disseminated between members of both groups. One school acted as the lead agency for the budget, an arrangement which worked well.

A budget allocation to buy out teacher's time during the school day would have been beneficial in developing long term curriculum and environmental changes.

This proved to be a beneficial mix in facilitating the communication between the support and evaluation team and the schools. In year one, the Project Coordinator was employed fulltime, which permitted the establishment of networks and collaborations between the schools and the community. In the subsequent years, the coordinator's time fraction was decreased to half time, which reduced the amount of face-to-face time and scope for working with the community. The workload of the SPOs exceeded the amount of allocated time, which impacted negatively on their classroom teaching during peak times when they had organised major activities and programs for their school.

This model of a Project Coordinator on varying time fractions and SPOs employed one day per week to drive this level of activity was probably not sustainable for long-term or wide-scale implementation of health promotion within schools. More central (e.g. state-level) coordination of curriculum development, social marketing messages and materials, and training programs along with central policy support for health-promoting schools is probably a more integrated, sustainable and efficient model.

#### Lessons learnt

• Project governance and management should incorporate representatives from the whole-of-community.

• Transparent reporting mechanisms are required.

The collection of process evaluation for the IYM project as reported in this paper differed from that of other projects reported in the literature [20-23] which generally related to a standardised intervention. In the IYM project, a set of intervention activities were not uniformly applied but were tailored to the specific needs and interests of each school. Consequently, the number, range and content of activities varied widely between schools, as did the personnel involved. Our process evaluation focused on dose, frequency and reach of the activities conducted, and did not cover issues such as fidelity [23], quality [21], satisfaction [20], and maintenance [20]. The experience of IYM provides some insights into the complexities of planning and implementing multi-site, multi-strategy obesity prevention interventions which are less structured, and organic and evolving in nature.

## Conclusions

Preventing obesity among adolescents will require a multi-faceted approach, including community-based initiatives, such as IYM, which utilise the knowledge, expertise and leadership available within schools from the principals, teachers and student leaders to plan, develop and implement policies, programs and activities to promote healthy eating and physical activity. Harnessing these resources requires not only a high level of motivation, energy and coordination at the school level but also policy support from the education sector and partnership with the health sector. Indeed, closer links between the health and education sectors and their joint ownership of health-promoting schools programs is probably a prerequisite for sustainable obesity prevention strategies for youth.

Peer-led approaches, such as the Student Ambassador Model, are also a promising way to contribute to a school environment and ethos which makes healthy eating and participation in physical activity the norm. Student involvement in the decision-making processes also helps to achieve changes, such as changing the canteen foods; such changes directly affect the students and may meet with some resistance.

Analyses of the effectiveness and cost-effectiveness of the IYM program will determine whether the interventions described in this paper were sufficient to slow the increase in adolescent body mass index in this demonstration project. However, irrespective of these outcome results, many process lessons have been learned [20-23] which will contribute to the overall efforts to prevent adolescent overweight and obesity.

## Competing interests

The authors declare that they have no competing interests.

## Authors' contributions

LM was the Project Coordinator and the lead writer; AS and MM oversaw the collection and analysis of the process evaluation data; BS was the senior researcher overseeing all aspects of the project. All authors contributed to the writing and interpretation of this work.

## Pre-publication history

The pre-publication history for this paper can be accessed here:

http://www.biomedcentral.com/1471-2458/10/448/prepub
